# Masticatory muscle tendon-aponeurosis hyperplasia that was initially misdiagnosed for polymyositis: a case report and review of the literature

**DOI:** 10.1186/s40902-023-00386-6

**Published:** 2023-05-01

**Authors:** Wataru Katagiri, Daisuke Saito, Satoshi Maruyama, Makiko Ike, Hideyoshi Nisiyama, Takafumi Hayashi, Jun-ichi Tanuma, Tadaharu Kobayashi

**Affiliations:** 1 Department of Oral and Maxillofacial Surgery, Gifu University Graduate School of Medicine, Yanagido, Gifu, 501-1194 Japan; 2 Division of Reconstructive Surgery for Oral and Maxillofacial Region, Faculty of Dentistry & Graduate School of Medical and Dental Science, Niigata University, 2-5274 Gakkocho-dori, Chuo-Ku, Niigata, 951-8514 Japan; 3 Oral Pathology Section, Department of Surgical Pathology, Niigata University Hospital, 1-754 Asahimachi-Dori, Chuo-Ku, Niigata, 951-8520 Japan; 4 Division of Oral and Maxillofacial Radiology, Faculty of Dentistry & Graduate School of Medical and Dental Sciences, Niigata University, 2-5274 Gakkocho-Dori, Chuo-ku, Niigata, 951-8514 Japan; 5 Division of Oral Pathology, Faculty of Dentistry & Graduate School of Medical and Dental Sciences, Niigata University, 2-5274 Gakkocho-dori, Chuo-Ku, Niigata, 951-8514 Japan

**Keywords:** Aponeurosis, Calcineurin, Hyperplasia, Masticatory muscles, Square-shaped mandible, Fascia, Magnetic resonance imaging, Myotomy, Polymyositis, Tendons

## Abstract

**Background:**

Masticatory muscle tendon-aponeurosis hyperplasia (MMTAH) is a relatively newly identified clinical condition that manifests as trismus with a square-shaped mandible. Herein, we report a case of MMATH that was initially misdiagnosed for polymyositis due to trismus and simultaneous lower limb pain, with literature review.

**Case presentation:**

A 30-year-old woman had a history of lower limb pain after exertion for 2 years. Initial physical examination had been performed at the Department of General Medicine in our hospital. There was also redness in the hands and fingers. Although polymyositis was suspected, it was denied. The patient visited our department for right maxillary wisdom tooth extraction.

Clinical examination revealed that the patient had a square-shaped mandible. The maximal mouth opening was 22 mm. There was no temporomandibular joint pain at the time of opening. Furthermore, there was awareness of clenching while working. Panoramic radiography revealed developed square mandibular angles with flattened condyles. Computed tomography showed enlarged masseter muscles with high-density areas around the anterior and lateral fascia. Magnetic resonance imaging also showed thickened tendons and aponeuroses on the anterior surface and inside bilateral masseter muscles. Finally, the patient was diagnosed with MMTAH. Bilateral aponeurectomy of the masseter muscles with coronoidectomy and masseter muscle myotomy was performed under general anesthesia. The maximum opening during surgery was 48 mm. Mouth opening training was started on day 3 after surgery. Histopathological examination of the surgical specimen showed that the muscle fibers were enlarged to 60 μm. Immunohistochemistry testing for calcineurin, which was associated with muscle hypertrophy due to overload in some case reports, showed positive results. Twelve months after surgery, the mouth self-opening and forced opening were over 35 mm and 44 mm, respectively.

**Conclusions:**

Herein, we report a case of MMATH. Lower limb pain due to prolonged standing at work and overload due to clenching were considered risk factors for symptoms onset of MMATH.

## Background

Trismus can occur because of various reasons, such as temporomandibular joint disorder, trauma, tumors, and inflammation [1].

Masticatory muscle tendon-aponeurosis hyperplasia (MMTAH) is a relatively newly identified clinical condition that manifests as trismus with a square-shaped mandible [2, 3]. The pathology of MMTAH is recognized as hyperplasia of the masseter muscle aponeurosis and temporalis muscle tendon [4]. However, the etiology and clinical aspects of this disease remain unknown.

We report a case of MMTAH in a patient who underwent detailed examinations for suspected polymyositis due to simultaneous lower limb pain and trismus at the Department of General Medicine.

## Case presentation

A 30-year-old female patient was referred to our hospital due to gingival swelling surrounding the upper right third molar. Her home dentist recommended tooth removal for trismus at the Department of Oral and Maxillofacial Surgery.

She did not have trismus because it slows progressed and was asymptomatic. A review of her medical history revealed that she had lower limb pain after exertion for 2 years that was initially noted at the Department of General Medicine. She also had a history of autism spectrum disorder.

**Fig. 1:**
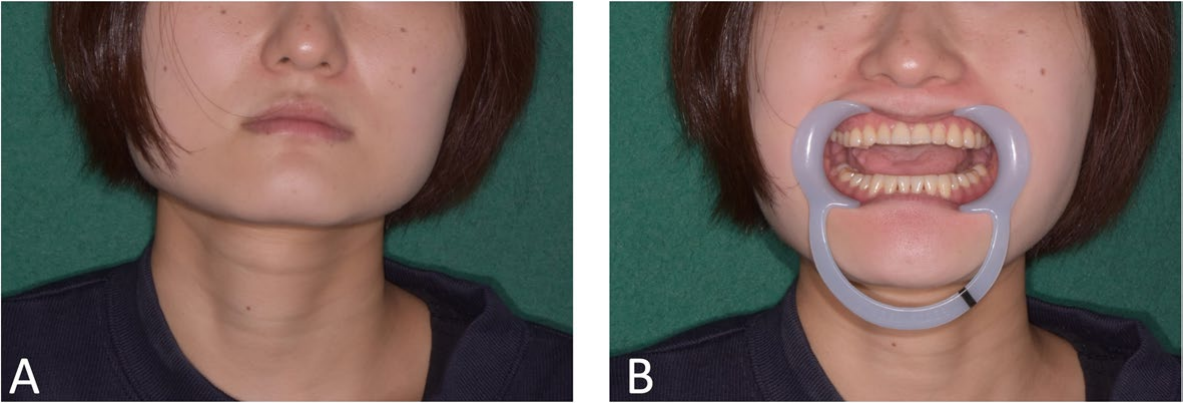
Extraoral photograph at first visit. Closed mouth (**A**) and maximum mouth opening (**B**)

**Fig. 2:**
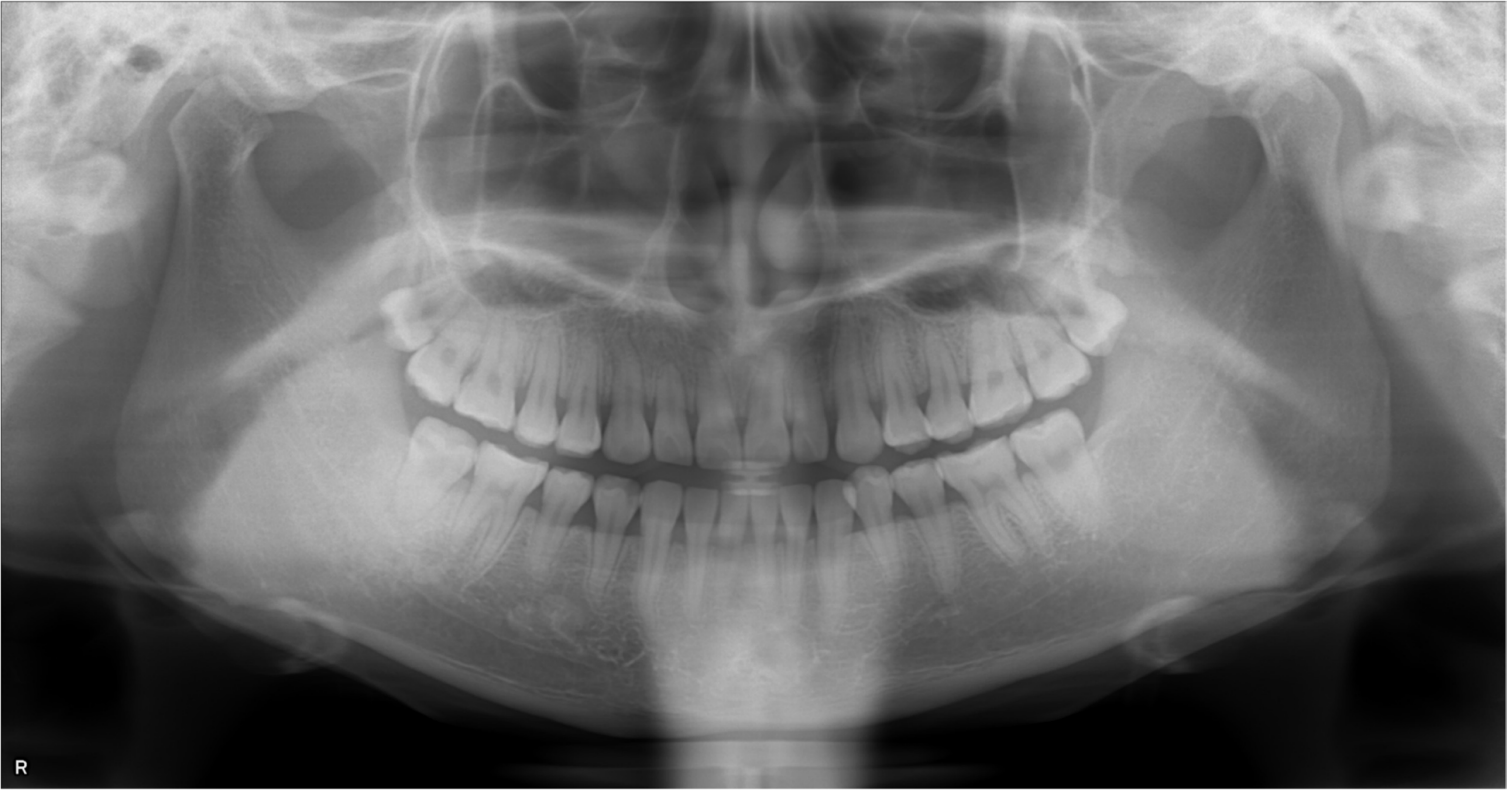
Preoperative panoramic X-ray. A panoramic radiograph showed a square-shaped mandible with a prominent mandibular angle and lower gonial angle

**Fig. 3:**
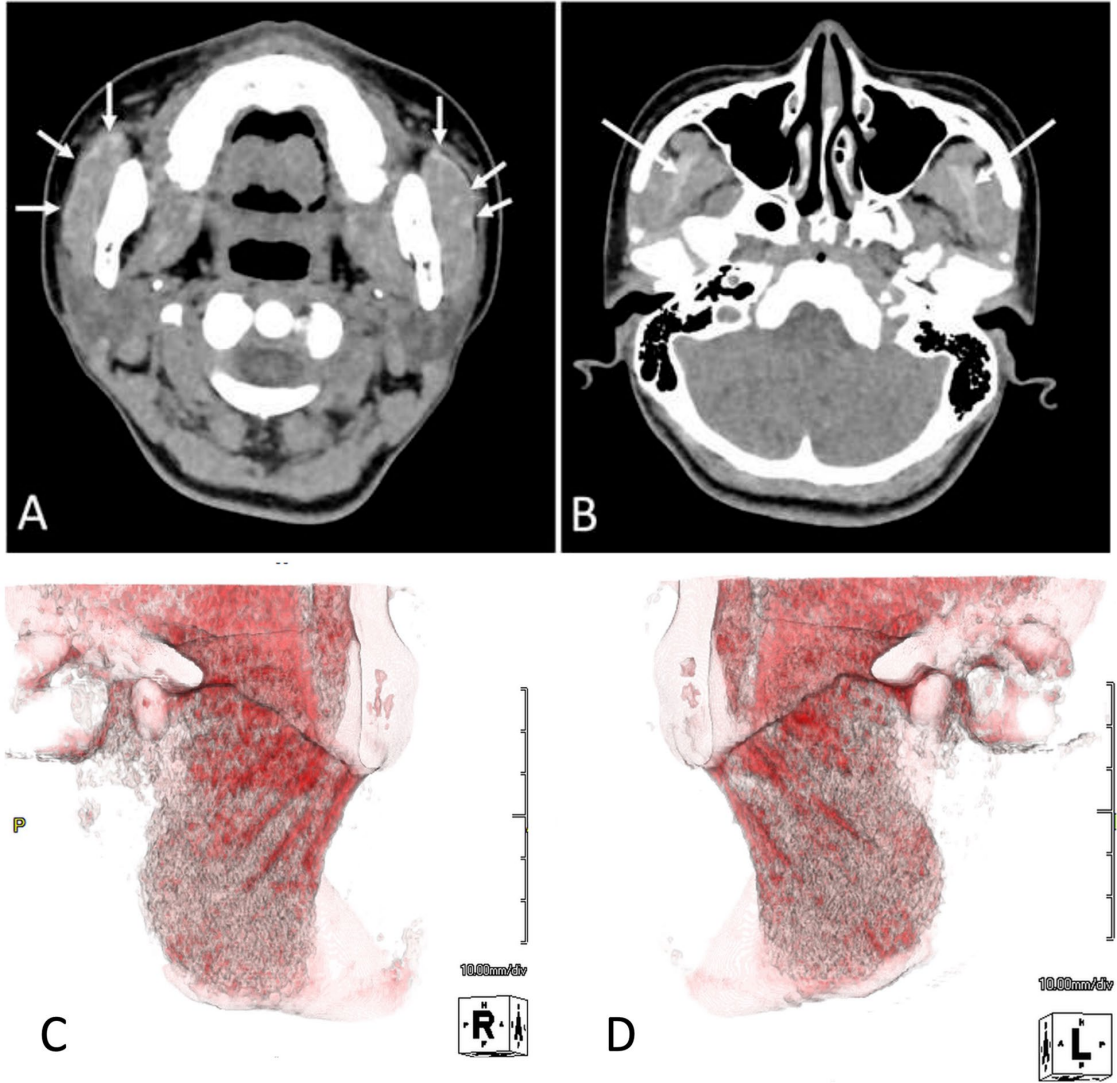
Preoperative images of computed tomography (CT). **A** Axial section of the masseter muscle. High-density areas are shown at the anterior and lateral part of the bilateral masseter muscle (arrows). **B** Bilateral temporal muscles are thickened at the level of infratemporal fossa, and high-density areas are also shown at the temporal muscle tendons and aponeuroses (arrows). **C** and **D** Three-dimensional volume-rendering CT images. Red color indicated higher CT value area at the anterior and lateral part of the bilateral masseter muscles compared with the average CT value of the masseter muscle

**Fig. 4:**
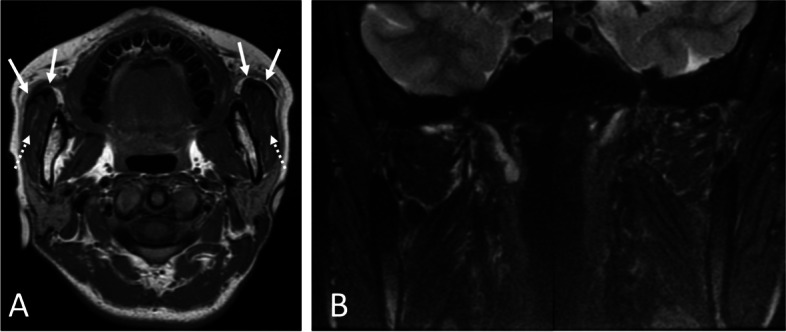
Preoperative magnetic resonance imaging (MRI). **A** Axial view of T1-weighted image (T1WI). Arrows show bilateral enlarged masseter muscle aponeurosis. Dotted arrows show high intensity areas in the masseter muscle. **B** Frontal views of T2-weighted images (T2WI). There is no evidence of inflammatory legions within the masticatory muscles

Clinical examination revealed that the patient had a square-shaped mandible. The maximal mouth opening was 22 mm (Fig. 1 A, B). Panoramic radiographs showed developed square mandibular angles with flattened condyles (Fig. 2). Computed tomography (CT) revealed enlarged masseter muscles with high-density areas in the anterior and lateral parts (Fig. 3 A–C). Magnetic resonance imaging (MRI) also revealed thickened tendons and aponeuroses on the anterior surface and inside both masseter muscles (Fig. 4 A, B). The patient was diagnosed with MMTAH.

**Fig. 5:**
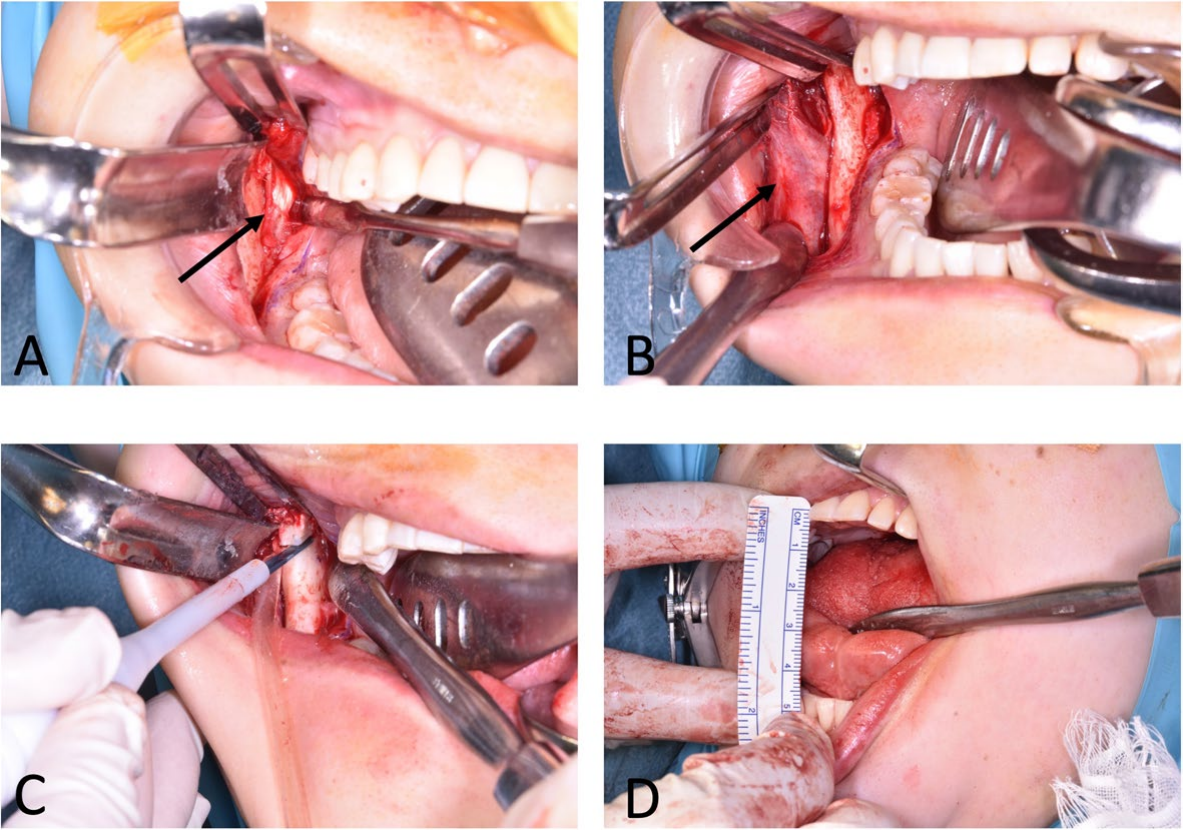
Photographs during surgery. **A** A white temporal tendon-aponeurosis lateral to the surface of the masseter muscle (arrow). **B** Masseter muscle and tendon-aponeurosis (arrow). **C** Coronoidectomy using an ultrasonic bone-cutting device. **D** Measurement of mouth opening during surgery

Bilateral aponeurectomy of the masseter muscles with coronoidectomy and masseter muscle myotomy were performed under general anesthesia. Before the procedure, the maximal mouth opening was 22 mm under the effect of muscle relaxant. After incision of the anterior margin of the ramus, firm fascia and hyperplastic aponeurosis of the temporal muscles were identified and partially resected (Fig. 5 A, B). Bilateral coronoidectomy was performed (Fig. 5C). Thereafter, the maximal mouth opening reached 38 mm. Then, the firm aponeurosis of the anterior part of the masseter muscles was partially resected. Additionally, the firm connection of the muscle to the mandibular angle was also released. Finally, a mouth opening of 48 mm was achieved, and the surgery was successfully completed (Fig. 5D).

**Fig. 6:**
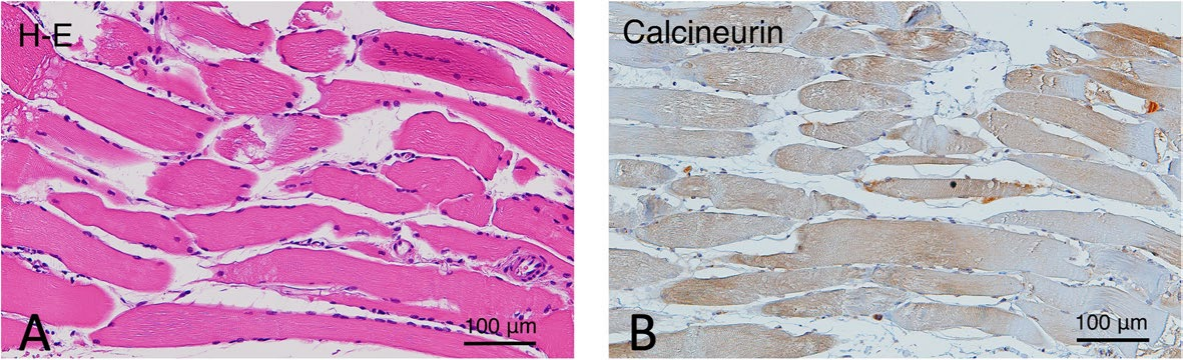
Histopathology of the masseter muscle. **A** H-E staining shows hypertrophic muscle fibers with an increase in their diameters between 20 and 60 μm. **B** Immunohistochemistry for calcineurin A. Calcineurin A expression is shown in the hypertrophic masseter muscles

Histopathological findings revealed that the surgical specimens from the masseter muscle had thick muscle fibers (Fig. 6A). The muscle cells showed hyperplasia and were immunohistochemically positive for calcineurin A (Fig. 6B). Finally, the patient was diagnosed with MMATH based on histopathological and clinical findings.

**Fig. 7:**
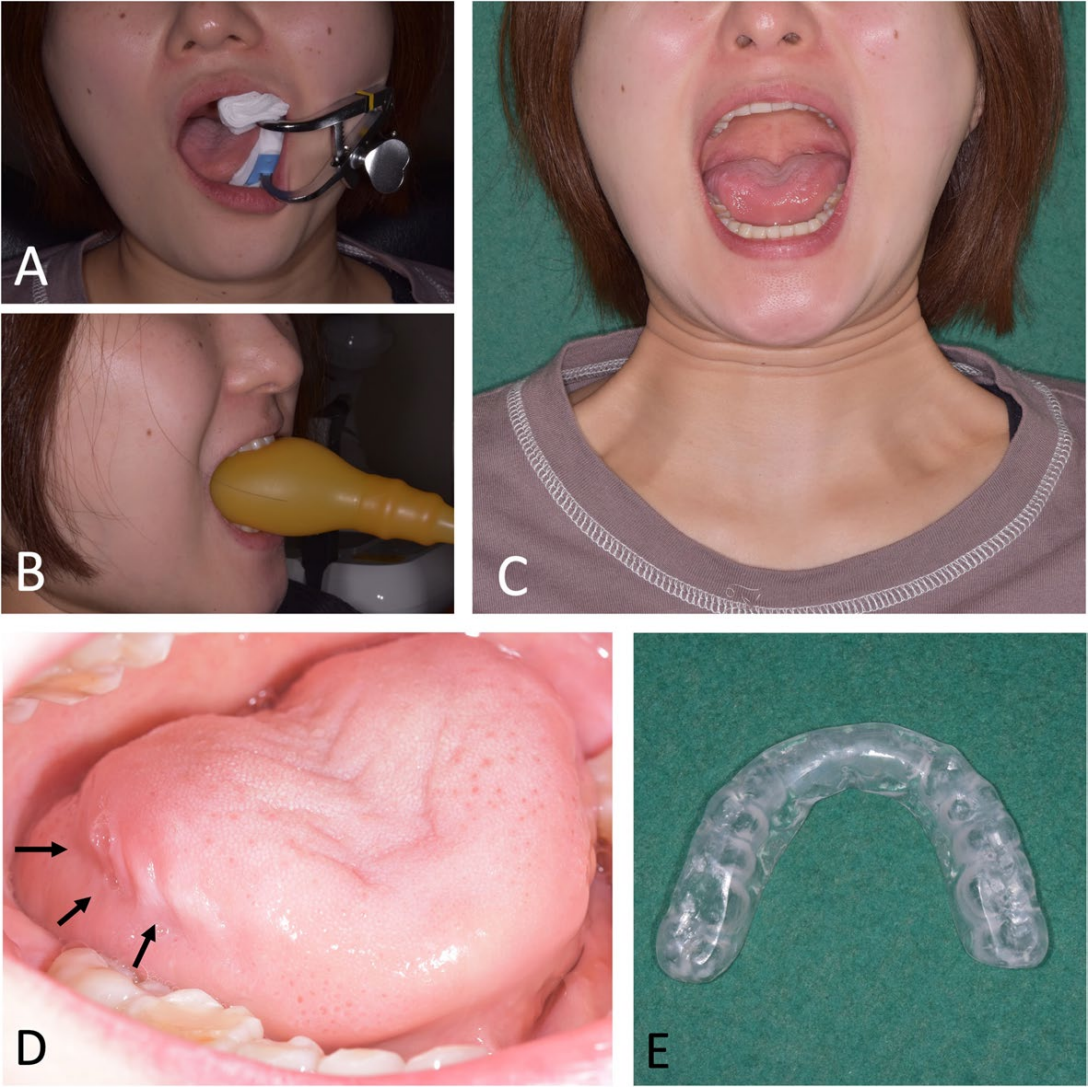
Postoperative follow-up. **A** Mouth-opening rehabilitation with a mouth gag at the clinic. **B** Self-rehabilitation with the insufflation-type mouth gag. **C** Maximum mouth opening 6 months after surgery. **D** Dental impression of the tongue (arrows). **E** Mouth guard used during her work time

Three days after the operation, mouth opening rehabilitation with a mouth gag was initiated. During the hospital stay for 1 week after surgery, rehabilitation was performed twice a day. After discharge, rehabilitation at the outpatient clinic was performed three times per week for 3 months. Rehabilitation was performed twice a week for 6 months after the operation (Fig. 7 A–C). Simultaneously, home rehabilitation using an insufflation-type mouth gag was performed every day (Fig. 7B). Since the patient also had a habit of clenching while working and there were dental impressions of her tongue, a mouth guard was also used during her office hours (Fig. 7 D, E).

**Fig. 8:**
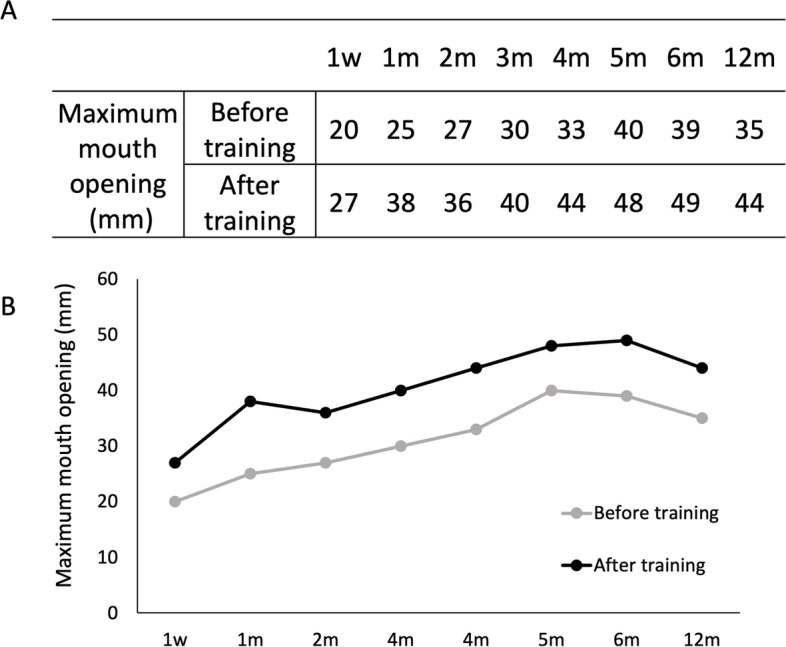
Mouth opening after surgery. **A** Amount of maximum mouth opening before and after training. **B** Graphical changes of maximum mouth opening at each period

The effects of mouth-opening rehabilitation were evaluated by measuring the interincisal distance every consultation day. The measurements performed at each month are shown (Fig. 8 A and B).

After discharge from the hospital, the mouth opening temporarily decreased. However, it gradually improved from approximately 4–6 weeks after the operation. At 6 and 12 months postoperatively, the maximum mouth opening was 49 mm and 44 mm, respectively (Fig. 8 A, B).

## Discussion

The Japanese Society of Temporomandibular Joints first defined and recognized MMTAH in 2008. MMTAH is a relatively newly identified disease entity characterized by trismus due to contracture of the masticatory muscles, resulting from hyperplasia of tendons and aponeuroses [2, 3]. According to previous reports, MMTAH is highly prevalent among female patients over a wide range of ages.

In this case, the patient was examined at the Department of General Medicine for suspected polymyositis due to simultaneous lower limb pain and trismus.

Although rheumatoid arthritis and scleroderma, classic connective tissue diseases, rarely cause severely limited mouth opening [5], limited mouth opening has been reported in patients with polymyositis and dermatomyositis. However, these reported cases were due to muscle inflammation, which spread to the surrounding tissue. In addition, histological findings in these cases showed chronic inflammation, fibrosis, and disappearance of muscle fibers [6, 7]. In this case, muscle inflammation was not found on CT or MRI. In addition, the administration of serum anti-Jo-1 antibody in this patient did not support the diagnosis of polymyositis.

Crincoli et al. also reported that bruxism, which was similar to the habit of clenching in this case, was not reported in patients with idiopathic inflammatory myopathies, such as polymyositis and dermatomyositis. They concluded that this could be explained by a condition of muscle weakness caused by loss of muscle mass and impaired intrinsic contractility [8].

The histopathological findings of this case revealed hyperplasia of thick muscle cells with diameters of approximately 60 μm, while normal cells were in the range of 20–40 μm as previously reported by Tsuneki et al. [9]. Masseter muscle hypertrophy is a relatively rare and benign enlargement of the unilateral or bilateral masseter muscles [10]. However, the etiology of masseter muscle hypertrophy remains under discussion. This asymptomatic persistent muscle enlargement has been reported to be initiated by bruxism, clenching, or heavy gum chewing [11, 12]. Although Martensson also indicated the relationship between the deformity and the so-called work hypertrophy [13], some reports have doubted this theory [14–16]. Beckers reported in his case reports that seven of 17 patients (41.2%) exhibited signs that seemed to support the work hypertrophy theory, such as bruxism and occlusal imbalances [17].

Work hypertrophy caused by exertion results in muscle enlargement. An increase in muscle fiber diameter from 20 to 60 μm has been reported to be the cause of increased masseter size [11, 18].

In our case, immunohistochemical findings revealed that these muscle cells were positive for calcineurin A, which has been reported to be relevant to muscle hypertrophy due to overload.

Studies using bite-opening rats proposed that calcineurin signaling [19], a calcium/calmodulin-regulated protein phosphatase that acts on the transcription factors of the nuclear factor of activated T-cell family, was an important molecular mechanism inducing masseter muscle hypertrophy [20].

Therefore, there might be a possibility that the overload of muscles of the master and lower limbs during her office time caused a series of symptoms in this patient.

To the best of our knowledge, 47 cases, from 5 reports, have been diagnosed with MMTAH and reported in English-language journals since 2008 [3, 21–24].

Coronoidectomy or coronoidotomy to remove the temporalis muscle tendon was performed to treat MMTAH in all cases. Aponeurectomy and excision of the masseter muscle were performed to remove hyperplastic tissue. However, mandibular anglectomy was performed in only three cases, according to these reports. This seems to be the case because it is now chosen for esthetic reasons, particularly for square mandibles [25, 26].

Botulinum toxin type A, which is a powerful neurotoxin produced by the anaerobic organism *Clostridium botulinum*, is one of the other treatment procedures for masseter muscle hypertrophy (MMH). Injecting botulinum toxin type A into a muscle causes interference with the neurotransmitter mechanism, producing selective paralysis and subsequent atrophy of the muscle [27–29]. However, there have been no reports on the use of botulinum toxin type A for treatment of MMTAH.

The biggest limitation of botulinum toxin therapy is that the treatment effect wears away within 6 months and the original condition recurs. Unlike surgical excision of muscular tissue, which reduces the actual number of muscle cells, botulinum toxin type A only temporarily reduces muscle volume [29]. Therefore, patients must be informed of the recurrence rate after the procedure.

Postoperative mouth-opening rehabilitation is an important factor for good prognosis. Using MRI at 1 year after surgery, Sato et al. revealed that the temporal muscle reattached to the resected stump of the bone [30]. Many studies have reported that mouth-opening training was performed for 6–24 months after surgery for MMH and MMTAH.

**Fig. 9:**
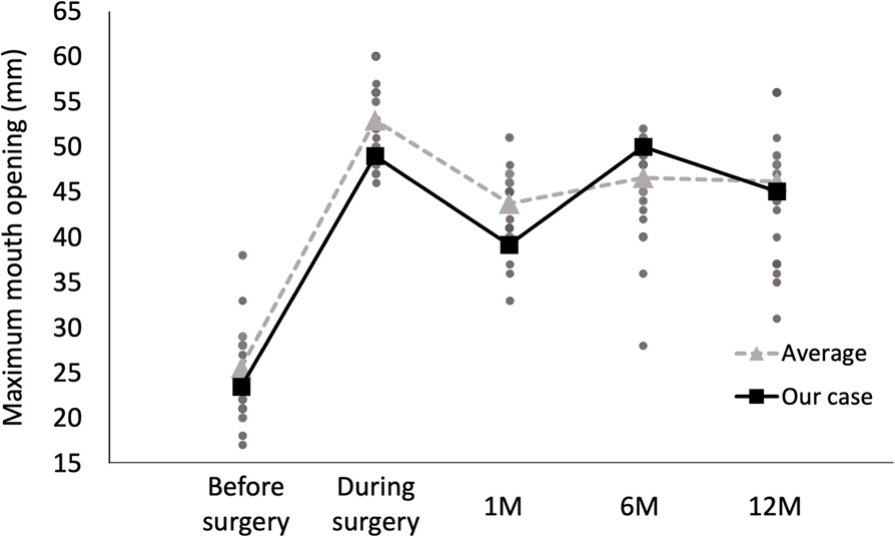
Summary of the mouth opening after the surgery from the previous reports. Gray dots indicate the data from 24 cases of maximum mouth opening before and during surgery and 1, 6, and 12 months after surgery. The gray line shows the average of these cases. The black dots and lines show the maximum mouth opening in each period in our case

In our case, because the patient visited our clinic constantly, the maximum mouth opening reached 48 mm 6 months and 44 mm 12 months after surgery using the mouth guard during her work time to remove negative factors, such as clenching, and obtain a more sufficient training effect (Figs. 7E and 8 A, B). Based on our review of previous reports, as far as we can summarize, the data from 24 cases of maximum mouth opening during the first year after surgery are shown including our case [3, 21–24]. Although the maximum mouth opening was reduced 1 month after surgery, it was thought to be due to an inflammatory reaction. However, the maximum mouth opening became stable 6 and 12 months after surgery (Fig. 9).

## Conclusion

A case of MMTAH is reported, in which polymyositis was first suspected at the Medical Department. Burden on the lower limbs due to prolonged standing at work and overload due to clenching were considered factors for causing the patient’s symptoms. Pathologically, the masseter muscle showed hyperplasia, which was suspected to be due to overloading. Clinically good long-term results were obtained after surgery with continuous mouth-opening rehabilitation.

## Data Availability

Not applicable.

## Abbreviations

MMTAH: Masticatory muscle tendon-aponeurosis hyperplasia
MMH: Masseter muscle hypertrophy
CT: Computed tomography
MRI: Magnetic resonance imaging
H-E: Hematoxylin and eosin
